# Rhinovirus replication and innate immunity in highly differentiated human airway epithelial cells

**DOI:** 10.1186/s12931-019-1120-0

**Published:** 2019-07-12

**Authors:** Stephanie M. Warner, Shahina Wiehler, Aubrey N. Michi, David Proud

**Affiliations:** Department of Physiology & Pharmacology and Snyder Institute for Chronic Diseases, Cumming School of Medicine, University of Calgary, 3280 Hospital Drive NW, Calgary, AB T2N 4Z6 Canada

**Keywords:** Highly differentiated human airway epithelial cells, Human rhinovirus, Negative strand template, Interferons, Viperin, Viral clearance

## Abstract

**Background:**

Human rhinovirus (HRV) infections are the primary cause of the common cold and are a major trigger for exacerbations of lower airway diseases, such as asthma and chronic obstructive pulmonary diseases. Although human bronchial epithelial cells (HBE) are the natural host for HRV infections, much of our understanding of how HRV replicates and induces host antiviral responses is based on studies using non-airway cell lines (e.g. HeLa cells). The current study examines the replication cycle of HRV, and host cell responses, in highly differentiated cultures of HBE.

**Methods:**

Highly differentiated cultures of HBE were exposed to initial infectious doses ranging from 10^4^ to 10^1^ 50% tissue culture-infective dose (TCID_50_) of purified HRV-16, and responses were monitored up to 144 h after infection. Viral genomic RNA and negative strand RNA template levels were monitored, along with levels of type I and II interferons and selected antivirals.

**Results:**

Regardless of initial infectious dose, relatively constant levels of both genomic and negative strand RNA are generated during replication, with negative strand copy numbers being10,000-fold lower than those of genomic strands. Infections were limited to a small percentage of ciliated cells and did not result in any overt signs of epithelial death. Importantly, regardless of infectious dose, HRV-16 infections were cleared by HBE in the absence of immune cells. Levels of type I and type III interferons (IFNs) varied with initial infectious dose, implying that factors other than levels of double-stranded RNA regulate IFN induction, but the time-course of HRV-16 clearance HBE was the same regardless of levels of IFNs produced. Patterns of antiviral viperin and ISG15 expression suggest they may be generated in an IFN-independent manner during HRV-16 infections.

**Conclusions:**

These data challenge a number of aspects of dogma generated from studies in HeLa cells and emphasize the importance of appropriate cell context when studying HRV infections.

## Introduction

Human rhinovirus (HRV) infections are responsible for over 50% of common cold-like illnesses [1], and HRV is the most common virus triggering acute exacerbations of asthma and chronic obstructive pulmonary disease [2–4].

The primary site of HRV infection and replication in both the upper and lower airways is the human airway epithelial cell [5, 6], and it is thought that HRV-induced changes in epithelial biology contribute to symptoms. In support of this, HRV-infected human airway cells, grown either as undifferentiated cells in submersion culture, or as cells differentiated by culture at air-liquid interface (ALI), release proinflammatory chemokines and cytokines, several of which are also detected in airway secretions during HRV infections [7, 8]. HRV infections also trigger epithelial induction of a range of potential host antiviral molecules that may limit viral replication [7, 9, 10].

Much of our understanding of how HRV replicates and induces host antiviral responses, is based on studies using non-airway cell lines (e.g. HeLa cells), or is inferred from studies of other picornaviruses. HRV replication requires virus entry into cells and genome release [11]. The viral polyprotein is then translated from the positive sense RNA genome and the viral RNA polymerase subsequently generates negative strand copies of the HRV genome. These negative strands serve as templates for replication of positive strand genomes for packaging in the virus capsid [12]. Thus, during RV replication, double-stranded RNA (dsRNA) is generated which, according to dogma, not only regulates levels of viral replication but also is recognized by pattern recognition receptors that mediate the induction of proinflammatory and antiviral responses [13]. Viral release from infected cells then occurs via cell lysis [12]. Although it is generally held that innate immunity plays a key role in regulating the outcome to HRV infections, it is thought that immune cells also play an important role in viral clearance [14].

Human airway epithelial cells can now be cultured to reproduce in vivo epithelial morphology, and can survive for months, permitting experiments of much longer duration than could be achieved with submersion cultures. We exposed highly differentiated cultures of human bronchial epithelial cells (HBE) to varying initial infectious doses of HRV and monitored responses for 144 h. We show that, contrary to existing dogma, HBE clear HRV infections without the need for immune cells. In addition, we observed that levels of dRNA, assessed by quantification of negative strand template RNA, correlate temporally and quantitatively with maximal intracellular viral genome loads. In marked contrast, maximal levels of type I and type III interferon (IFN)s, as well as of some other host defense molecules, are not related to peak negative strand template levels but, rather, vary depending on the initial infectious dose of HRV used.

## Methods

### Isolation of human bronchial epithelial cells

HBE were obtained by protease digestion of dissected airways (main stem bronchus to 4th generation) as previously described [15], and stored in aliquots in liquid nitrogen until used. Each “n” value for experimental data represents the use of cells from a different individual donor.

### Air-liquid Interface culture of HBE cells

HBE cells were cultured on T75cm^2^ flasks (Costar, Corning Inc., Corning, NY) in Bronchial Epithelial Growth Medium (BEGM, Lonza, Walkersville, MD) supplemented with 5% FBS for 72 h (Life Technologies, Burlington, Ontario, Canada). Cells were then fed every 48 h with BEGM without FBS. At 90% confluence, cells were lifted and seeded at 2.0 × 10^5^ cells per insert onto 1.12cm^2^, 0.4 μm pore transwell inserts (Costar) coated with bovine collagen Type I/III (Advanced BioMatrix, San Diego, CA), and cultured in BEGM for 48 h. BEGM was then removed and HBE were cultured using only basolateral PneumaCult-ALI differentiation medium containing 100X supplement, hydrocortisone, and heparin (Stemcell Technologies, Vancouver, BC, Canada), as well as fluconazole (Sigma-Aldrich, Oakville, Ontario, Canada) and penicillin/streptomycin (Life Technologies). Cells were fed basolaterally every 48 h. Beginning 14 days after seeding, cells were washed apically once per week with PBS to remove excess mucus. Cultures were used for experiments at 5 weeks after transwell seeding.

### Viral propagation of HRV-16

HRV-16 stock was propagated in WI-38 cells and purified by sucrose density centrifugation as described [16]. Purified HRV-16 was dialysed, using a 10,000 kDa membrane, against 25 mM HEPES/F12 media for 18–20 h at 4 °C to remove sucrose. Replication-deficient HRV-16 was generated by exposure to Ultraviolet light for 5 min as described [17].

### Human rhinovirus inoculation

ALI cultures were infected apically with HRV-16 diluted in 25 mM HEPES/F12 at 34 °C for 4 h. Cells were then washed apically 5 times with PBS to remove non-internalized HRV-16 and maintained at 37 °C, feeding basally with PneumaCult every 24 h. Shed HRV-16 was collected at appropriate time points by rinsing the apical surface of ALI cultures with 500 μL PBS. Intracellular RNA was isolated for assessment of intracellular HRV levels.

### Immunofluorescence staining and microscopy

ALI cultures were fixed in 4% paraformaldehyde (Alfa Aesar, Heysham, UK) for 15 min at room temperature. Cells were blocked and permeabilized with 10% BSA, 5% NGS, 5% normal rabbit serum, 5% normal mouse serum in PBS (0.1% TritonX-100) for 2 h and rinsed in PBS. Cells were stained with mouse anti-β-tubulin (Sigma) at 1:200 in 2% BSA in PBS (0.1% TritonX-100) overnight at 4 °C. Cells were washed 3 times with PBS (0.1% TritonX-100) and stained with α-mouse AlexaFluor488 (ThermoFisher Scientific, Waltham, MA) at 1:200 in 5% BSA in PBS (0.1% TritonX-100) for 2 h at room temperature. Cells were washed 5 times with PBS (0.1% TritonX-100) and re-blocked with 10% mouse serum in PBS for 3 h. Mouse anti-dsRNA antibody (Scicons, Szriak, Hungary) was labeled with an AlexaFluor555 labeling kit (ThermoFisher), pre-absorbed at 1:200 in 5% BSA, 5% normal mouse serum in PBS for 1 h, and added to cells overnight at 4 °C. Cells were washed 3 times with PBS (0.1% TritonX-100) and nuclei subsequently stained with DAPI (ThermoFisher) at 1:50,000 for 20 min. Inserts were mounted using FluorSave (EMD Millipore) and imaged on a Leica TCS SP8 resonant scanning confocal microscope (Leica, Wetzlar, Germany) with Leica LasAF software.

### Histology

HBE were fixed in 10% neutral buffered formalin, embedded in paraffin, and sectioned to 4 μm thickness onto Superfrost plus slides. Alcian blue and hematoxylin staining was performed by de-paraffinization in two changes of xylene and rehydrating through graded ethanol solutions (100, 95, 70% EtOH). Alcian blue 8GX (Sigma) in 3% acetic acid solution was added for 2 min and rinsed in water for 2 min. Hematoxylin Gills II (Leica Biosystems) staining was performed for 5 min and rinsed in warm tap water for 5 min. Slides were dehydrated through reverse graded ethanol solutions (95, 100% EtOH) and cleared in two changes of xylene before applying coverslip with Permount (ThermoFischer).

### RNA extraction and real-time qRT-PCR

Total cellular RNA was isolated with the NucleoSpin RNA kit (Macherey-Nagel GmbH & Co, Duren, Germany), which included homogenization of the lysates, an on-column DNase digestion and elution in RNase/DNase free water. RNA concentration and purity was determined on a NanoDrop 2000 spectrophotometer. HRV-16, viperin, CXCL10, ISG15, IFNλ1 and IFN-β mRNA expression were assessed by real-time RT-PCR using specific primers and a TaqMan probe for each gene. For each mRNA, a synthetic first-strand cDNA was used to generate standard curves to permit absolute quantification. Data were expressed as femtograms calculated from the standard curve. For HRV-16, data were converted to copy number. For positive sense genomic RNA, the detection limit of the RT-PCR system corresponded to a total copy number of 10^6^.

Viral RNA from apical wash samples were isolated with the QIAamp Viral RNA Mini Kit (Qiagen, Mississauga, Ontario, Canada) and subjected to quantitative RT-PCR using primers and probe directed to the 5′-untranslated region of HRV-16.

### Negative Strand template primer design

This assay uses a chimeric primer for reverse transcription containing a non-viral sequence at the 5′ end of the specific sequence targeting the negative strand of HRV-16. This non-viral sequence is then used as a primer to selectively amplify the negative strand specific cDNA at the PCR stage. We validated that this assay was selective using synthetic, HPLC-purified negative and positive strand oligonucleotide sequences that spanned the sequences used for the assay. We confirmed not only that the primer system for negative strand did not show a positive signal when used with an excess of positive strand, but also that that detection of serial dilutions of negative strand was not affected by the presence of a multi-fold excess of positive strand sequence.

One microgram of RNA was reverse transcribed with Maxima reverse transcriptase with RNase H+ activity (Invitrogen, Carlsbad, CA) and the chimeric primer (Table 1). The non-viral sequence was taken from within the *Escherichia coli* genome and a Blast search confirmed the lack of any homology within the rhinovirus genome. To remove any potential carry over RNA, cDNA was RNase treated with an RNase cocktail (Invitrogen). cDNA was then purified using the QIAquick PCR Purification Kit (Qiagen, Hilden, Germany). Real-time PCR was carried out with a HRV-16 specific forward primer and probe, directed within the VP1 region of HRV-16, and the non-viral reverse primer. A synthetic first-strand cDNA standard was used to quantify the amount of template present. Absolute values were used to determine copy number.

**Table 1:** Primer and probe sequences used for the negative strand HRV-16 RT-PCR reactions

Name	Purpose of oligonucleotide	Sequence
Neg-Tag-cDNA	Chimeric RT primer	5′- ATCAGCGATGCCGAACGTAT GGCAGCATGGGCAACCT-3′
Neg-HRV16-For	Real-time PCR Forward Primer	5′-TGCTGATGCAATACTCAAAAAGG-3’
Tag-Rev	Real-time PCR Reverse Primer	5′- ATCAGCGATGCCGAACGTAT -3’
Neg-Probe	Real-time PCR Probe	5’FAM-TGAAAAGCGAGGGA-MGB3’

Underlined nucleotides denote non-viral tag sequences

### Elisa

CXCL10 was measured by ELISA using matched antibody pairs (PeproTech, Rocky Hill, NJ). IFN-λ1 and IFN-β ELISAs were from R&D Systems (Minneapolis, USA).

## Results

### Epithelial cultures

**Fig. 1:**
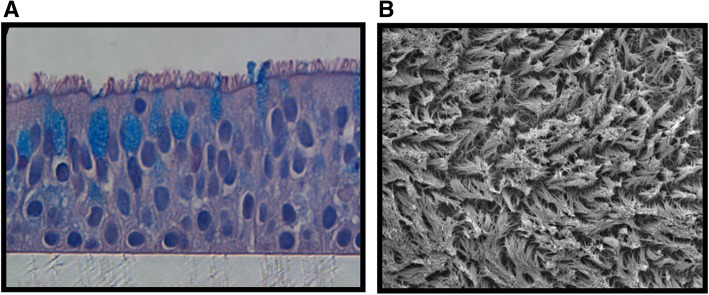
Morphology of highly differentiated human bronchial epithelial cultures. **a** Histology of HBE cultures showing a pseudostratified morphology with numerous cilia. Goblet cells are shown using Alcian blue staining. **b** Scanning electron micrograph demonstrating a dense blanket of cilia

HBE cultured at air-liquid interface (ALI) for 5 weeks using PneumaCult-ALI differentiation medium show a morphology that resembles bronchial epithelium in vivo with a pseudostratified columnar structure containing well-defined goblet cells and numerous ciliated cells (Fig. 1). These 5-week cultures show an increased height and greater cilia coverage compared to typical 3-week cultures (not shown).

### HRV-16 infection does not cause overt cell toxicity in HBEs

**Fig. 2:**
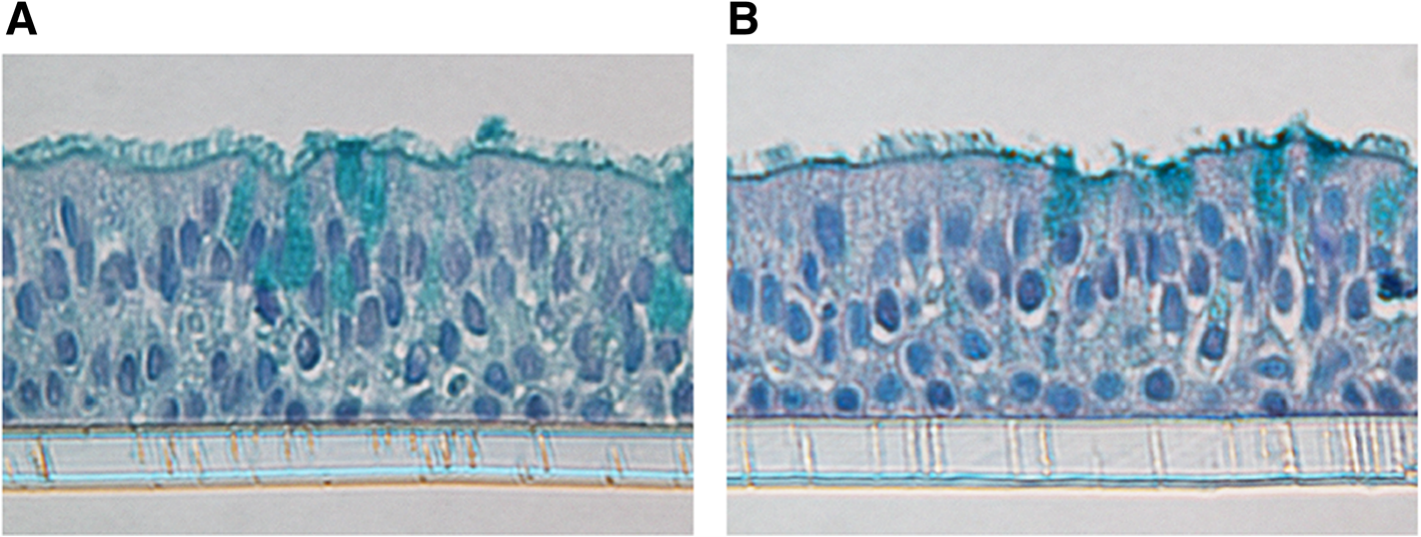
HRV-16 infection does not cause overt epithelial damage. Histology of HBE cultures at 144 h after exposure to **a**) medium control or **b**) 10^4^ TCID_50_ of HRV-16

Highly differentiated cultures of HBE showed no evidence of overt toxicity at any point during infection. Histological evaluation of cultures exposed to either medium or to 10^4^ TCID_50_ of HRV-16 when studied after a 144 h infection period (Fig. 2), showed no obvious differences, either between the two treatments or when compared to basal 5 week cultures (Fig. 1a).

### HRV genomic RNA levels in response to varying infectious doses of RV-16

**Fig. 3:**
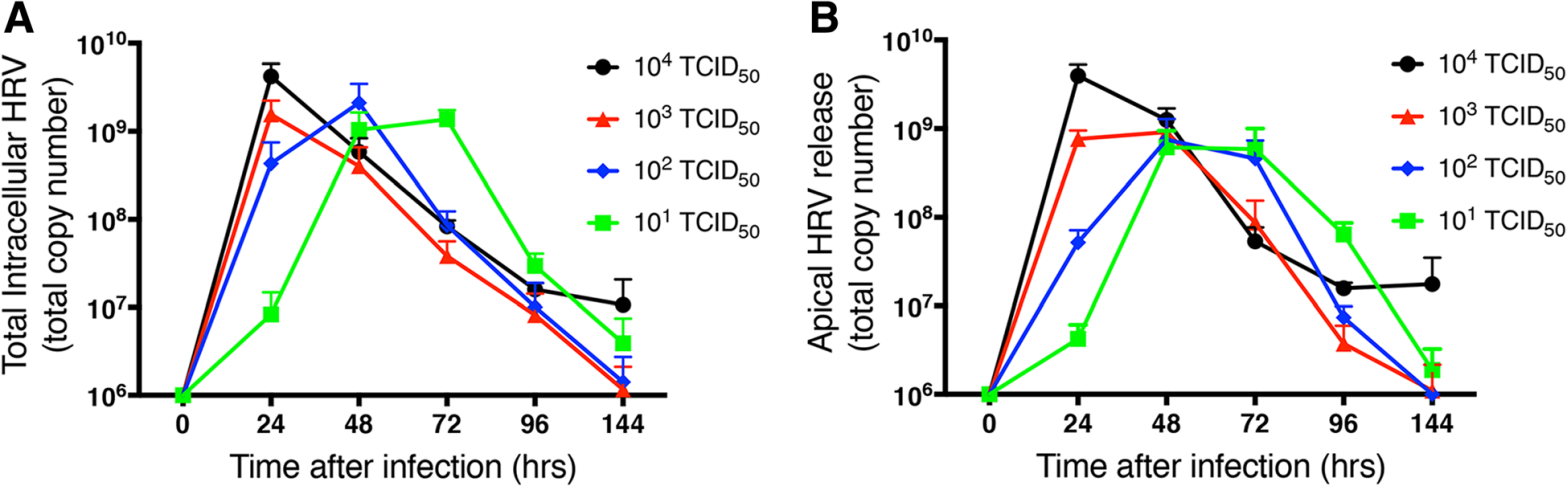
Rhinovirus replication and clearance in highly differentiated HBE. Cells were infected with varying initial doses of HRV-16. **a** Total intracellular HRV genomic RNA levels with time. **b** Total levels of apically shed HRV genomic RNA with time. Data are mean ± SEM from 4 different HBE donors. The limit of detection corresponded to a total copy number of 10^6^

HBE from 4 different lung donors were infected with doses ranging from 10^4^ to 10^1^ 50% tissue culture-infective dose (TCID_50_) of HRV-16, and intracellular and shed levels of HRV genomic RNA were monitored with time by RT-PCR and expressed as total copy number (either in all cells from an insert or as total amount released). The timing of peak levels of intracellular genomic RNA varied, occurring later with lower infectious doses (Fig. 3a). Despite this, the peak copy number of genomic HRV observed was similar regardless of initial infectious dose. Interestingly, by 144 h after infection, HRV viral genome within the cells had declined to undetectable levels at all infectious doses except 10^4^ TCID_50_, where detection of residual burden was limited to a single donor.

HRV shedding from infected HBE occurred exclusively at the apical surface, with no detectable virus in basolateral medium. For each initial infectious dose, the time course of peak levels of HRV shedding mirrored those for intracellular virus, suggesting a “steady-state” between intracellular virus and the rate of shedding (Fig. 3b). Again, shed virus levels returned to undetectable by 144 h post-infection, except for the same single donor infected with 10^4^ TCID_50_ of HRV-16. To confirm that virus measured by RT-PCR reflected infectious virions, HRV in apical washes from cells infected with an initial dose of 10^4^ TCID_50_ was measured by viral titer assay and showed the same pattern of viral load (not shown).

### Generation of negative strand viral RNA

**Fig. 4:**
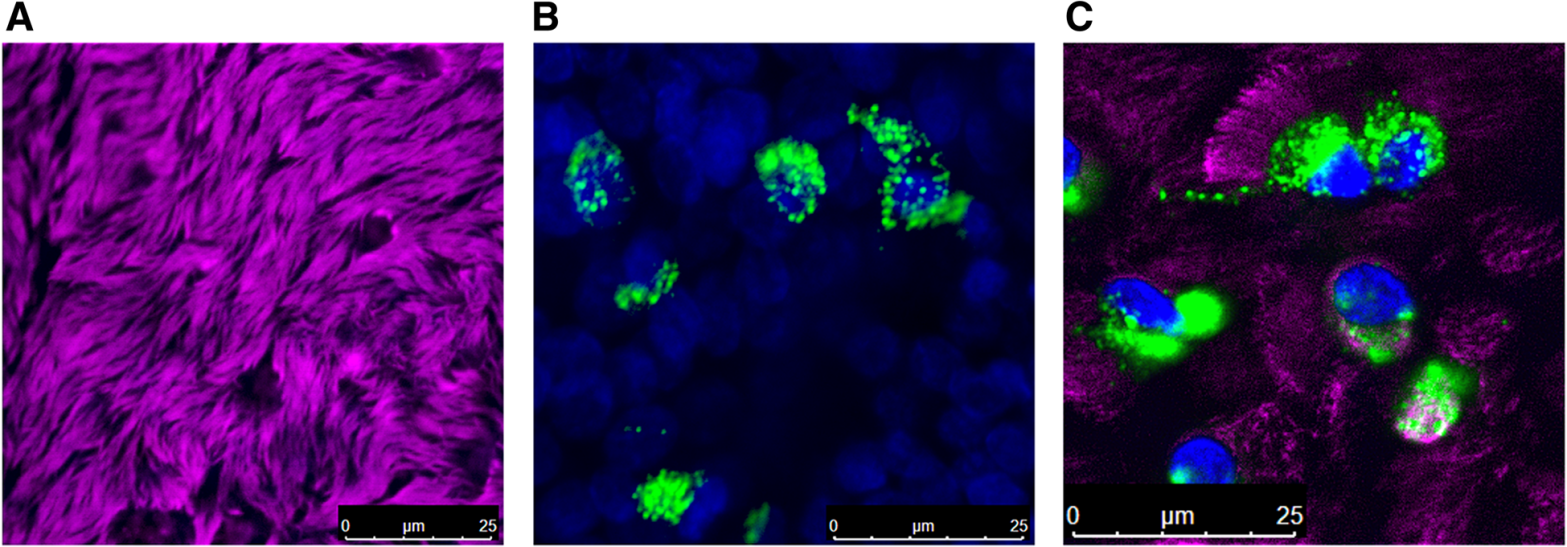
Immunofluorescence staining of dsRNA in HBE infected with HRV-16. **a** Staining with an antibody to β-tubulin (violet) shows dense cilia on cultures of HBE. **b** Intracellular perinuclear staining for dsRNA (green) 24 h after infection with 10^4^ TCID_50_ of HRV-16. Nuclei are stained blue with DAPI. **c** Co-staining of cilia (violet) and dsRNA (green) shows viral infection and replication occurs in ciliated cells. Scale bars are shown on each panel. Data are representative of *n* = 4

To understand the relationship of dsRNA to HRV replication and innate antiviral immunity, we initially performed immunofluorescence confocal microscopy on our densely ciliated cultures (Fig. 4a) with a monoclonal antibody directed to dsRNA [18] using cells exposed to 10^4^ TCID_50_ of HRV-16. No fluorescence signal was observed in non-infected HBE or in HBE exposed to replication-deficient HRV-16 (not shown). In cells infected with HRV-16, perinuclear dsRNA staining was observed 24 h post infection (Fig. 4b). Interestingly, staining was restricted to ciliated cells (Fig. 4c). To determine percentage of total cells positive for dsRNA, cells from each of 4 donors were used, and three different fields of view for each donor were assessed. A reticule was used to define fields of view and percentage of total cells in the field (based on DAPI nuclear staining) that were positive for dsRNA was recorded. Using this method an average of only 4–6% of total cells showed positive staining for dsRNA.

**Fig. 5:**
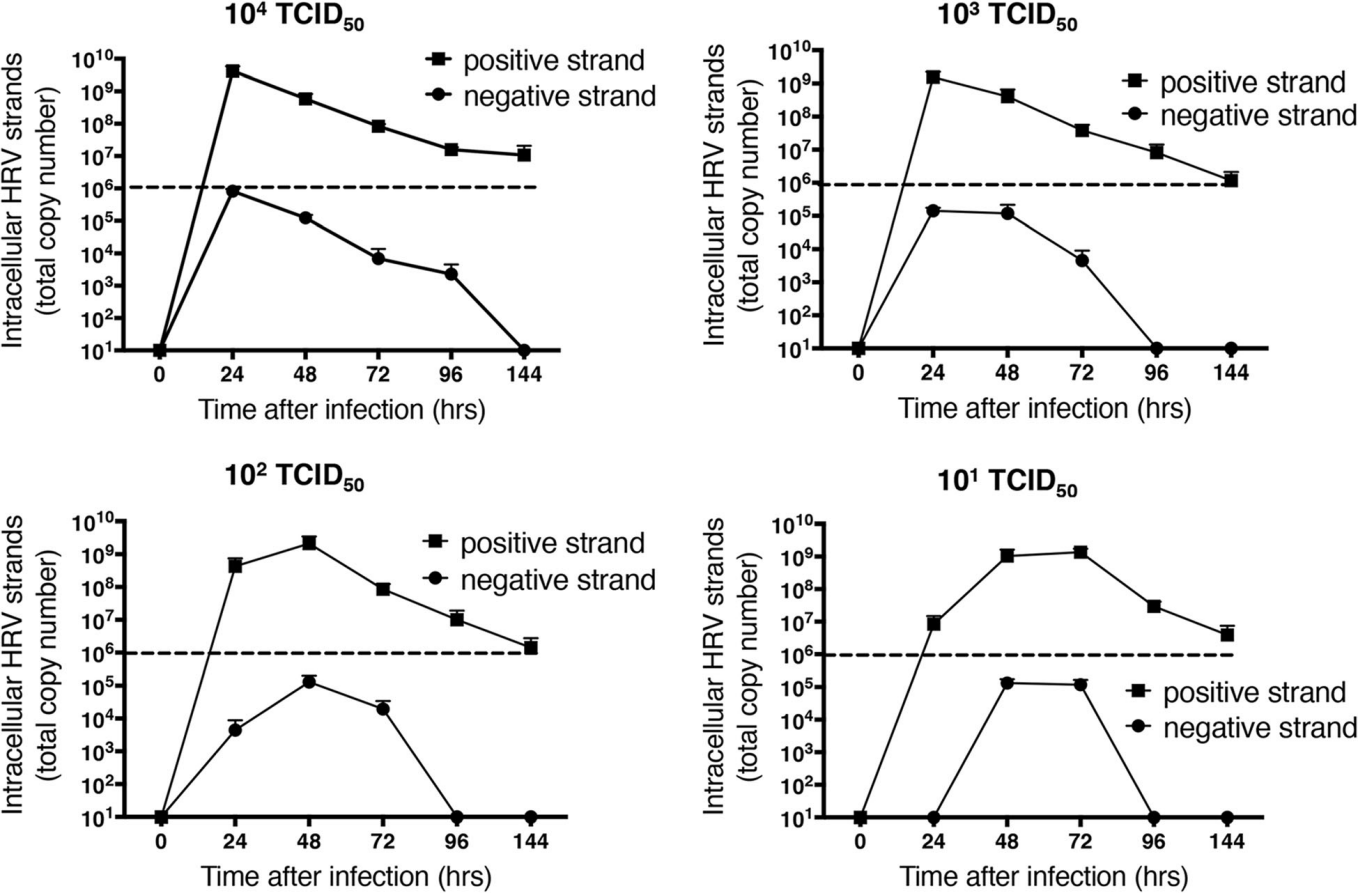
Comparison of total copy numbers HRV-16 intracellular positive (genomic) and negative (template) RNA strands at multiple times after infection with varying levels of HRV-16. Data are mean ± SEM from 4 different HBE donors. The dotted horizontal line shows the limit of detection for the RT-PCR for genomic RNA

We next developed an assay to specifically quantify negative strand template RNA, expressed as total copy number, as an index of levels of dsRNA. The temporal pattern of negative strand template production with initial infectious dose closely mirrored levels of positive strand genome (Fig. 5). As was seen for positive strand, similar peak intracellular levels of negative strand RNA were observed regardless of the initial dose of virus used for infection. However, in all cases, levels of negative strand were approximately 10,000-fold lower than those of positive strand genome.

### Relationship of dsRNA to production of host defense molecules

**Fig. 6:**
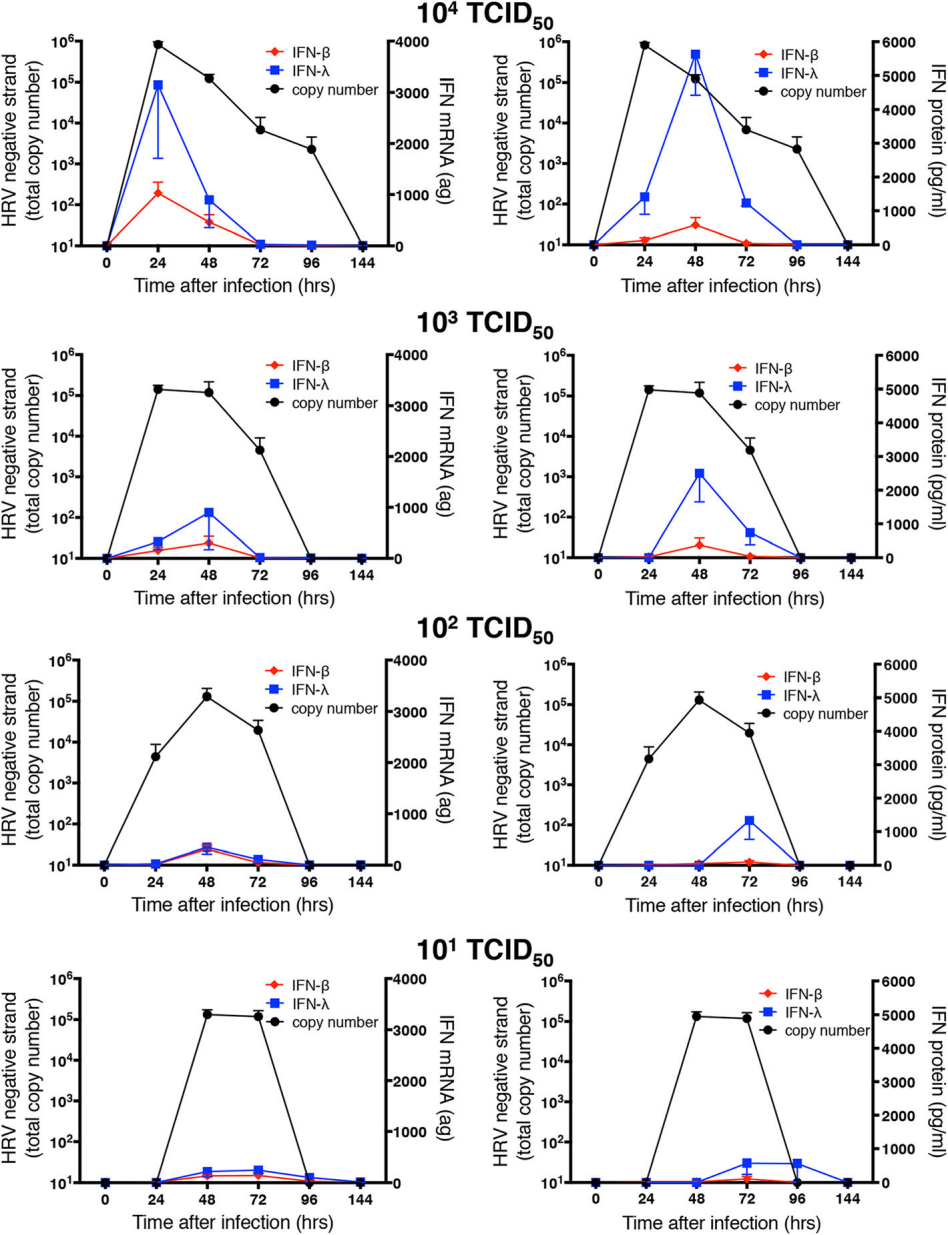
Relationship between copy number of negative strand template (taken from Fig. 5) and mRNA or protein for IFNs. For each initial infectious dose of HRV-16, copy number of negative stand template is shown relative to levels of IFN-λ1 (blue) or IFN-β (red). Left panels show IFN mRNA and right panels show apically secreted protein levels. Data are mean ± SEM from 4 different HBE donors

**Fig. 7:**
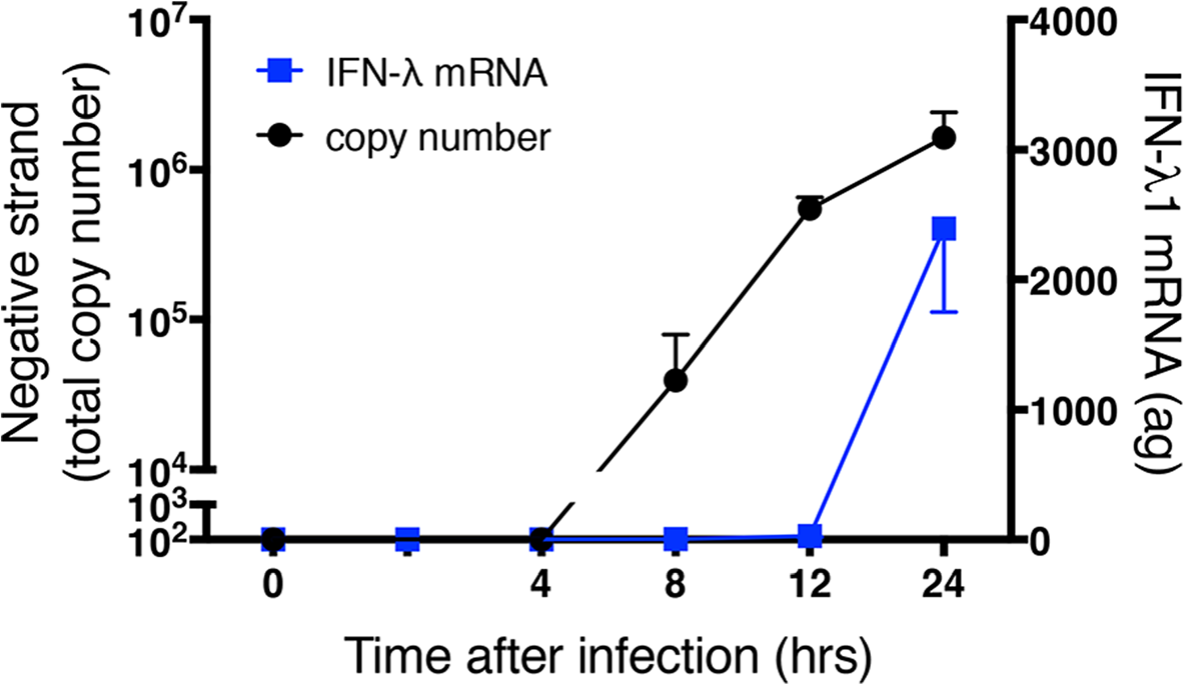
Negative strand copy number and levels of mRNA over the first 24 h after infection with 10^4^ TCID_50_ of HRV-16. Data are mean ± SEM from 4 different HBE donors

We then used the same negative strand copy number data to assess the relationship of dsRNA levels to production of a number of other molecules from the same cultures. In contrast to the close association between levels of negative strand template and genomic positive strand, no such relationship was observed for production of IFNs. Levels of intracellular mRNA for IFN-λ1 and IFN-β, were temporally related to those of negative strand template (Fig. 6), but did not show a quantitative relationship. Instead, levels of IFN mRNA declined with the initial infectious dose of HRV-16 used. Levels of secreted IFN-λ1 and IFN-β proteins, which were released dominantly at the apical surface, showed a similar pattern, although, depending on infectious dose of HRV, peak protein secretion occurred later than maximal mRNA levels (Fig. 6). Because levels of negative strand were already maximal at 24 h following infection with 10^4^ TCID_50_ of HRV-16, we performed separate experiments over periods up to 24 h after infection with this dose of HRV-16. Negative strand template was detectable by 8 h after infection and increased to peak levels at 24 h (Fig. 7), Interestingly, increased expression of IFN-λ1 mRNA was not observed until 24 h, when it was already comparable to the maximal level observed in Fig. 6.

**Fig. 8:**
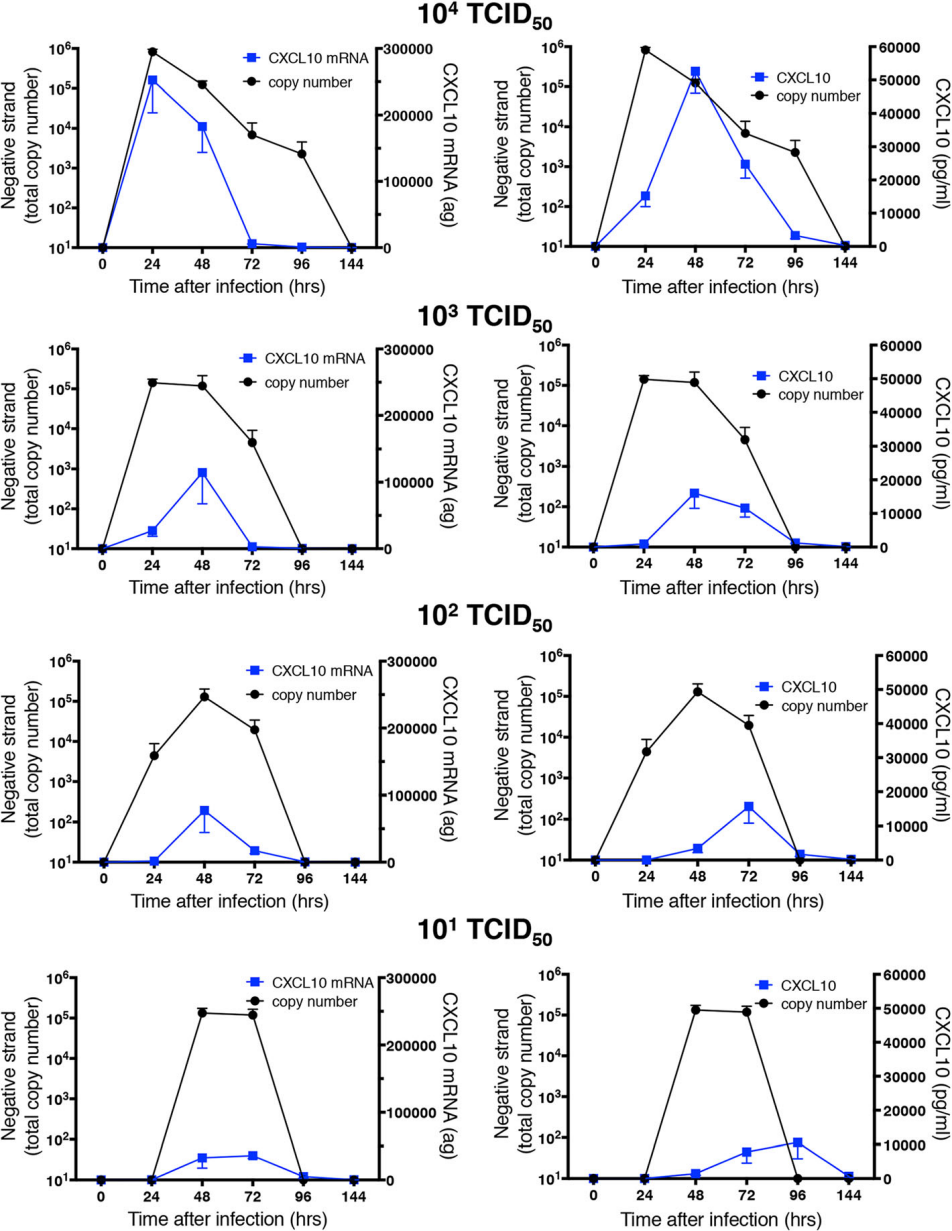
Relationship between copy number of negative strand template (taken from Fig. 5) and mRNA or protein for CXCL10. For each initial infectious dose of HRV-16, copy number of negative stand template is shown relative to levels of CXCL10 mRNA (left panels) mRNA or basolateral protein release (right panels). Data are mean ± SEM from 4 different HBE donors

**Fig. 9:**
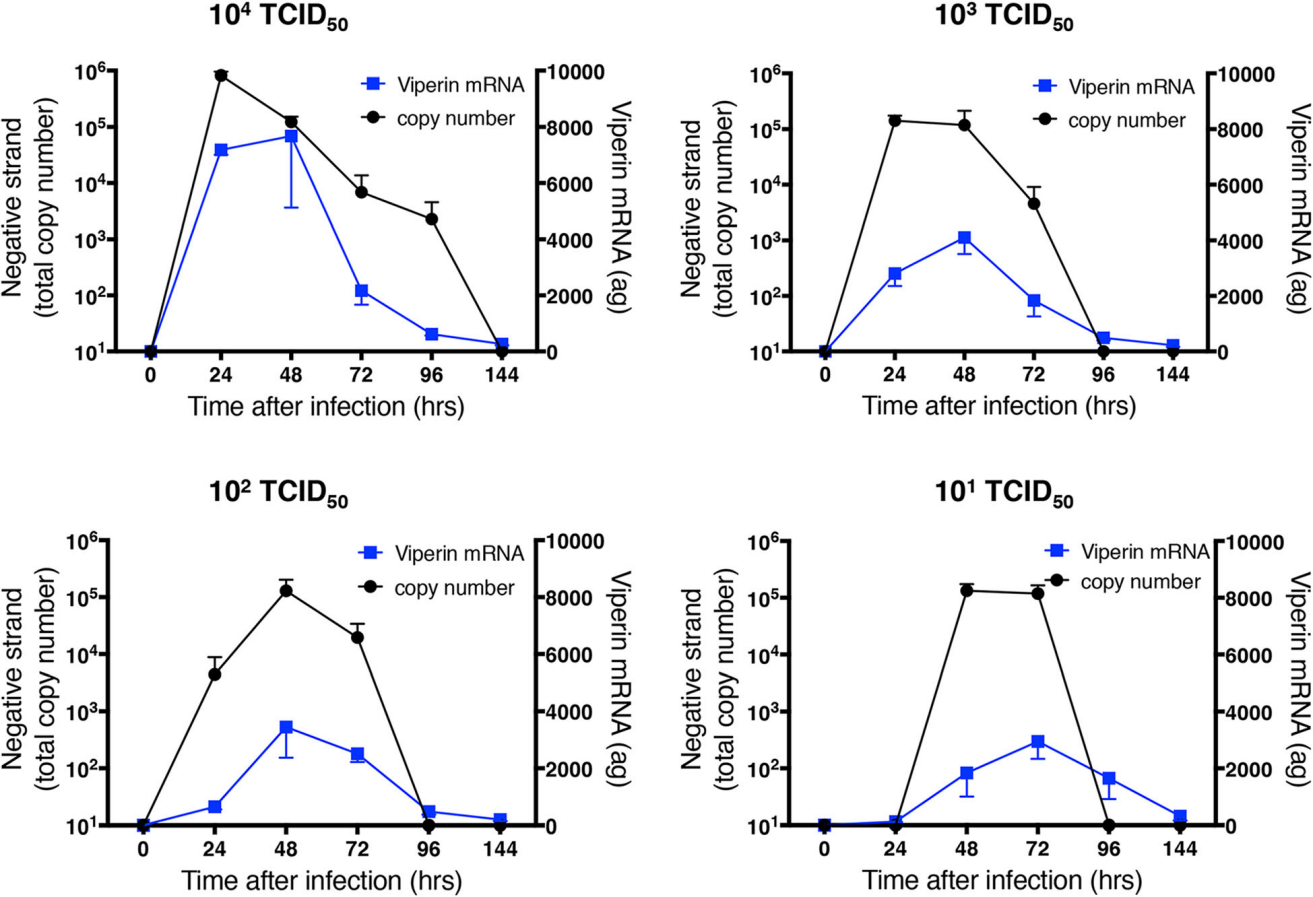
Relationship between copy number of negative strand template (taken from Fig. 5) and mRNA for viperin. For each initial infectious dose of HRV-16, copy number of negative stand template is shown relative to levels of viperin mRNA Data are mean ± SEM from 4 different HBE donors

**Fig. 10:**
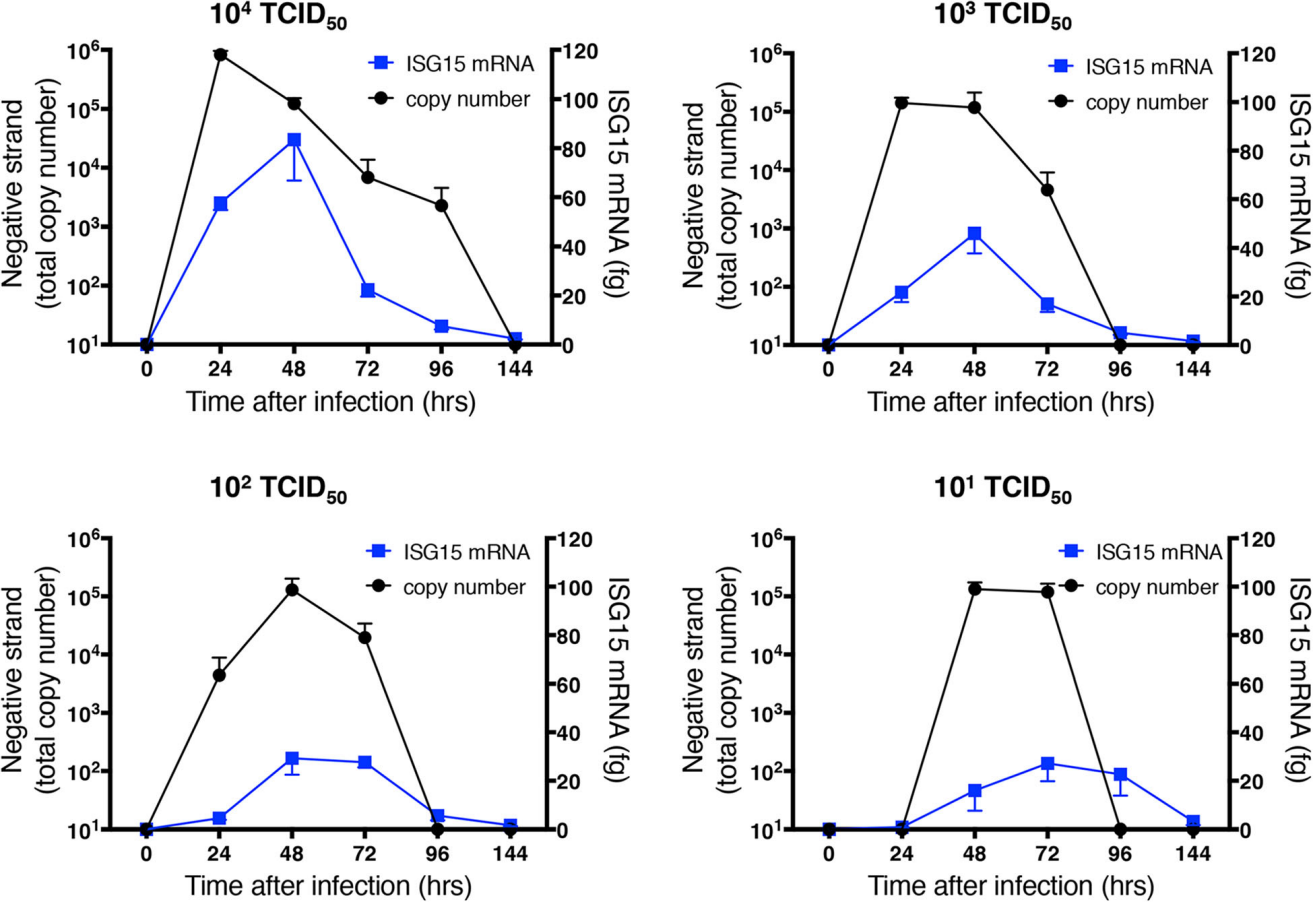
Relationship between copy number of negative strand template (taken from Fig. 5) and mRNA for ISG15. For each initial infectious dose of HRV-16, copy number of negative stand template is shown relative to levels of ISG15 mRNA Data are mean ± SEM from 4 different HBE donors

Expression of mRNA and protein secretion (predominantly into the basolateral medium) of the chemokine CXCL10 showed similar temporal and quantitative patterns to the IFNs, with a clear relationship between levels of induction and initial infectious dose (Fig. 8). We have previously shown roles for viperin and ISG-15 in host defense to HRV infection [9, 19], so also examined mRNA expression for each of these molecules (Figs. 9 and 10). At the highest initial infectious dose (10^4^ TCID_50_ of HRV-16), peak expression of both viperin and ISG15 was slightly delayed relative to those of IFNs and CXCL10. Moreover, the dependence on infectious dose was less clear, as comparable viperin expression was observed at all initial infectious doses (except 10^4^ TCID_50_ of HRV-16), and the reduction of ISG15 expression at lower doses was less marked. Generation of all of the host defense molecules examined depend upon viral replication, since exposure of HBE to 10^4^ TCID_50_ of HRV-16 that was rendered replication deficient by exposure to UV light did not induce epithelial expression of any of the molecules studied (not shown).

## Discussion

The HeLa cervical carcinoma cell line was invaluable for the development of vaccines against poliovirus [20], and the ease of infection of HeLa cells by picornavirus family members has led to the frequent use of these cells for studies of picornavirus replication. The physiological relevance of such studies of HRV replication must be questioned, however, since HRV selectively infects the airways. HRV infection studies in vivo demonstrate that epithelial cells are the natural site of HRV infection and replication [5, 6, 21], and the use of human airway epithelial cell lines [22], and subsequently primary HBE grown in submersion culture [23, 24], or at ALI [25, 26], have provided important insights into the inflammatory and innate immune responses to HRV infection. HBE in submersion culture are not suited, however, for studying the complete HRV replication cycle, as confluent cultures cannot be maintained for extended periods. Initially, ALI cultures of HBE used cells grown in “bronchial epithelial differentiation medium” for 3 weeks yielded cultures that were multi-layered and expressed some cilia but did not reproduce the in vivo pseudostratified columnar morphology [25, 26]. By contrast, 5-week ALI cultures grown in PneumaCult medium recapitulate in vivo morphology. Moreover, these cultures can be maintained for months at ALI, permitting the current studies examining the HRV replication cycle in response to varying initial infectious doses of HRV. Although well differentiated cultures of human airway epithelial cells have been used to study kinetic responses to infection with respiratory syncytial virus [27, 28], to our knowledge this is the first use of such cells for extended kinetic studies of responses to HRV infection.

Our data clearly show that similar peak intracellular and shed levels of HRV genomic RNA are observed regardless of the initial infectious dose of virus, although the peak of viral replication was delayed at lower infectious doses. Whether the peak levels of intracellular (and shed) virus observed with all infectious doses represent the maximal viral replication that can be supported by the cells is unclear. Given that the initial infectious material removed from the cells contains levels of HRV that are not distinguishable from the dose applied (not shown), it would appear that few virions are taken up by cells. Thus, it is unclear why a delay in replication is observed with lower doses. One could speculate that fewer cells are initially infected with lower doses and that it takes longer for all cells that can support replication to be infected. Regardless, a major observation from the current studies is that highly differentiated HBE can clear HRV-16 infection by 144 h after infection, regardless of the initial infectious dose used. This is reflected both by intracellular and shed virus levels. It should be noted that the general time course of shed virus in these cultures resembles that observed in nasal secretions following experimental HRV-16 infection of normal volunteers [17]. Overall, these data indicate that epithelial antiviral responses on their own are adequate to clear HRV-16 infections and that, while recruitment of inflammatory and immune cells to the airway mucosa in vivo may contribute to symptoms, these cells are not essential for virus clearance.

The generation of negative strand template is a prerequisite for transcription of new genomic RNA strands. The process of transcribing negative strands to genomic RNA leads to the formation of dsRNA. To examine the formation of dsRNA we used two complementary approaches. Immunofluorescence staining with a validated antibody to dsRNA showed perinuclear staining of dsRNA at 24 h post infection (the time of maximal intracellular virus load). This perinuclear localization is consistent with the concept that HRV replicates on membrane fragments derived from endoplasmic reticulum or golgi [29, 30].

Using β-tubulin as a marker for cilia, we observed that HRV replication was restricted to ciliated cells. This agrees with recent reports that both HRV-A and HRV-C strains selectively infect ciliated cells [31, 32]. Our assessment that 4–6% of total cells were positive for dsRNA staining is consistent with the observed focal, patchy infection observed in bronchial biopsies [21], and with the report that only 5–10% of HBE grown in submersion culture can be infected regardless of the initial infectious dose of HRV used [33].

To our knowledge there has been no prior quantitative assessment of the level of dsRNA achieved in airway epithelial cells infected with HRV. We examined absolute copy numbers of negative strand as a means to quantify dsRNA. Prior studies of single strand genome viruses have shown that conventional RT-PCR targeting the negative strand does not provide strand-specificity. The phenomenon of “false priming” yields signals even in the absence of primer during reverse transcription [34, 35], and studies using single strands generated by in vitro transcription from plasmids have shown that RT-PCR for negative strand is not specific in the presence of excess positive strand [36]. We therefore modified a previously published strategy [36], in which the primer for reverse transcription contains a non-viral sequence at the 5′ end of the specific sequence targeting the negative strand of HRV-16. This non-viral sequence is then used as a primer to selectively amplify the negative strand specific cDNA at the PCR stage. This technique showed that similar levels of negative strand (and, thus, presumably, dsRNA) were achieved regardless of the initial infectious dose, albeit with varying time-courses. This implies that a defined level of negative strand is required for optimal replication of genomic RNA. If the concept put forward for poliovirus that genomes that have not been translated cannot function as templates for negative strand synthesis [37, 38] also holds true for HRV, this would suggest that a defined level of virus polyprotein strands are translated after infection in order to support optimal viral replication. Given that copy numbers of negative strand were about 10,000-fold lower than those of positive strand, transcription of positive strand from template must be highly efficient. Our data differ from a previous report of a ratio of only about 70 positive strands per negative strand in cells infected with poliovirus, assessed using an RNase protection assay [39]. This difference may be due to specificity of the natural cell host (the cell type used in the prior poliovirus study was never defined), differences between viruses, or the sensitivity and specificity of the methods used.

The dsRNA generated during viral replication is recognized by pattern recognition receptors (PRRs) to induce innate immune responses. Studies have reported varying roles of the dsRNA receptors, toll-like receptor-3, retinoic acid inducible gene-I and melanoma differentiation associated gene-5 in inducing epithelial responses to HRV infection [19, 40, 41]. Type I and type III IFNs are both produced by epithelial cells in response to viral infections but recent studies have indicated that type III IFNs, particularly IFN-λ1, are the dominant IFNs produced by human airway epithelial cells in response to HRV infection [10, 42]. We confirmed this observation in highly differentiated epithelial cells, showing that induction of mRNA and protein for IFN-λ1 are considerably higher than levels observed for IFN-β. Interestingly, however, despite the relatively constant level of negative strand template generated with each initial infectious dose of HRV-16, levels of mRNA and protein for type I and type III IFNs decreased markedly as the initial infectious dose of HRV decreased. Interestingly, levels of IFNs produced had no bearing on the time to HRV clearance, implying that neither of these types of IFNs control viral clearance. No IFNs were generated in cells exposed to HRV rendered replication deficient by exposure to UV light (not shown). Since HRV treated in this way can still engage its receptor, this implies that induction of IFNs requires viral replication and not just engagement of ICAM-1, the receptor for HRV-16. For each infectious dose of HRV-16, the kinetics, but not the quantity, of IFN induction mirrored that of negative strand template. Thus, the degree of induction of IFNs is not simply regulated by levels of dsRNA, as reflected by levels of negative strand. This implies that other factors generated during the replication cycle must play a role. The identity of these factors is unknown but single-stranded RNA, other replications intermediates, or other components of the viral genome, may contribute. Induction of CXCL10 mRNA and protein closely mirrored the induction of IFNs, both in terms of kinetics and quantity. Since we have previously reported that induction of CXCL10 by HRV-16 is not mediated via IFN signaling [17], it may be that induction of both CXCL10 and IFNs occur via shared pathways. It is of interest that there was a different vectoriality of release for IFNs (mainly apical) and CXCL10 (mainly basolateral). We have also observed this for other epithelial mediators (not shown). At this point the reasons for these differences are unknown.

Interestingly, although viperin and ISG-15 mRNA levels were highest when infected with 10^4^ TCID_50_ of HRV-16, levels were relatively constant at the three lower infectious doses. The persistence of mRNA expression for these two genes at lower infectious doses, despite the profound decline in IFN levels, suggest that their induction is not mediated by type I or type III IFNs. Although both viperin and ISG15 are usually referred to as members of the group of “IFN-stimulated genes” (ISGs), there is precedent that ISGs can be induced independently of IFNs. Such IFN-independent induction occurs via direct viral induction of key transcription factors, particularly members of the interferon regulatory factor (IRF) family. Thus, cells deficient in both type I and type III IFN receptors still showed induction of multiple ISGs in response to influenza infection [43]. Similarly, it has previously been show that viperin can be induced independently of IFN signaling via an IRF-1 mediated mechanism [44]. Consistent with this, we have previously shown robust induction of IRF-1 and IRF-7 in HRV-infected human airway epithelial cells [45, 46], and have demonstrated that induction of ISG15 by HRV is dependent upon transcriptional control by IRF-1 [19].

Although textbooks frequently state that HRV virions escape cells via lysis, this is based on studies in HeLa cells. It has been known for years, however, that HRV infections in vivo do not cause overt epithelial cytotoxicity [47]. Consistent with this observation, no overt cell death was observed in our highly differentiated cultures of HBE upon infection with HRV and an intact epithelium was still observed at 144 h after HRV infection (Fig. 2). Histological integrity was not different compared to cells exposed to medium for 144 h, or to epithelium prior to infection (Fig. 1a). We cannot rule out that death or shedding of a small percentage of cells occurs during infection, but if so, the epithelium either generates replacement cells or else adjacent cells expand to maintain an intact epithelium. In terms of alternative means for HRV to escape epithelial cells, there is precedent for HRV and other enterovirus species to leave other cell types via phosphatidylserine rich membrane vesicles with properties of either exosomes or autophagosomal like vesicles [48–50]. Additional studies will be required, however, to evaluate if this occurs in HRV infected highly differentiated HBE.

Our current studies used HRV-16, a member of the HRV-A genetic group of rhinoviruses that uses ICAM-1 to gain cell entry. We used HRV-16 because it is representative of a large number of HRV-A strains that also use ICAM-1, and has been commonly used for in vivo experimental infections. Nonetheless, we must avoid generalizing our data to all strains of HRV. Additional studies will be required to determine if HRV-A strains that use the low density lipoprotein as a receptor, or HRV-C strains that require cadherin related family member 3 to gain cell entry [51], show the same patterns of virus clearance, epithelial integrity, negative strand template expression and antiviral gene expression observed for HRV-16. It will also be of interest to determine if responses to a second consecutive infection, either with the same HRV strain or a different one, will show altered responses.

## Conclusions

In summary, we demonstrate that HRV-16 infects a subset of ciliated cells in highly differentiated cultures of HBE. These HRV-16 infections are cleared without the need for immune cells, and the time to clearance does not depend on levels of IFNs. Regardless of initial infectious dose used, relatively constant levels of genomic and negative strand RNA are generated, albeit with varying kinetics. Copy numbers of negative-strand RNA are some 10,000-fold lower than numbers of positive (genomic) strands. Although relatively constant levels of negative strands are generated regardless of initial infectious dose, levels of type I and type III IFNs vary depending upon initial infectious dose, implying that factors other than levels of dsRNA regulate IFN induction. Patterns of viperin and ISG15 expression suggest they may be generated in an IFN-independent manner during HRV-16 infections. These data challenge a number of widely held paradigms generated from earlier studies in HeLa cells and emphasize the importance of appropriate cell context when performing experiments using HRV infections.

## Data Availability

The authors’ unpublished data are available upon request.

## Abbreviations

ALI: Air-liquid interface
CXCL10: C-X-C chemokine ligand 10
dsRNA: double-stranded RNA
HBE: Human bronchial epithelial cells
HRV: Human Rhinovirus
IFN: Interferon
IRF: Interferon regulatory factor
ISG: Interferon stimulated gene
ISG15: Interferon stimulated gene of 15 kilodaltons
TCID_50_: 50% tissue culture-infective dose
